# On the Capability of Oxidovanadium(IV) Derivatives to Act as All-Around Catalytic Promoters Since the Prebiotic World

**DOI:** 10.3390/molecules25133073

**Published:** 2020-07-06

**Authors:** Patrizio Campitelli, Marcello Crucianelli

**Affiliations:** 1 School of Science and Technology, University of Camerino, via S. Agostino 1, 62032 Camerino (MC), Italy; patrizio.campitelli@unicam.it; 2 Department of Physical and Chemical Sciences, University of L’Aquila, Via Vetoio, Coppito-Due, 67100 L’Aquila (AQ), Italy

**Keywords:** vanadium oxides, prebiotic life, astrobiology, bioinorganic chemistry, catalytic oxidation, oxidovanadium complexes, acylpyrazolone ligands

## Abstract

For a long time the biological role of vanadium was not known, while now the possibility of using its derivatives as potential therapeutic agents has given rise to investigations on their probable side effects. Vanadium compounds may inhibit different biochemical processes and lead to a variety of toxic effects and serious diseases. But, on the other hand, vanadium is an essential element for life. In recent years, increasing evidence has been acquired on the possible roles of vanadium in the higher forms of life. Despite several biochemical and physiological functions that have been suggested for vanadium and notwithstanding the amount of the knowledge so far accumulated, it still does not have a clearly defined role in the higher forms of life. What functions could vanadium or its very stable oxidovanadium(IV) derivatives have had in the prebiotic world and in the origins of life? In this review, we have briefly tried to highlight the most useful aspects that can be taken into consideration to give an answer to this still unresolved question and to show the high versatility of the oxidovanadium(IV) group to act as promoter of several oxidation reactions when coordinated with a variety of ligands, including diketones like acylpyrazolones.

## 1. The Role of Oxidovanadium(IV) Derivatives or Vanadium Oxides in Prebiotic Chemistry

The name vanadium for the element with atomic number 23 was assigned in 1831 by Nils Gabriel Sefström, after the Scandinavian goddess of beauty and fertility, Vanadis (Freya) to underline the essential features of the rich and versatile vanadium chemistry.

It represents the second most abundant transition element in sea-water with a concentration of 30 nM (the first one is molybdenum, 100 nM) and it is distributed ubiquitously in soil, fossils, in crude oil, water, and air, both in soluble (dihydrogen-orthovanadate(V) [H_2_VO_4_^−^] anion) and insoluble (mainly oxidovanadium(IV) [VO]^2+^ cation) forms. Vanadium is also bioavailable for living organisms, where it looks like a redox couple H_2_VO_4_^−^/VO^2+^ (E = −0.34 V; pH = 7), allowing an easy change of the oxidation state [1,2].

But how could this element have been involved in the prebiotic world and in the origins of life? In a more general approach, we should focus our attention on the primary role played by metals and their simple derivatives like sulfides and oxides, as templating agents and catalytic promoters, during the key stages of the prebiotic evolutionary processes. This concept led to the term “metallome”, meaning a list of life supporting metals (among others Ca, Mg, Ni, V, Zn, Mo, Cu, W apart from the almost ubiquitous Fe) having a relevant role in several biological functions, thus driving the evolution [3]. The metallome did not just help primordial life to perform its basic functions but it also helped it in adapting to a changing Earth. For example, the coming of the Great Oxidation Event which transformed aqueous soluble ferrous salts to insoluble ferric salts, when oxygen levels rose to near modern levels, in the atmosphere. Consequently, in a more oxygen-rich atmosphere, copper salts became soluble allowing the metal ion hemocyanin to be bioavailable. Hemocyanin, the important copper-containing protein responsible for oxygen transport in *Mollusca* and *Arthropoda* [4], represents proof of how copper redox properties affected the availability of this metal in organisms. However, while the bioactivity of copper-containing proteins is well known, the same cannot be said for the bioinorganic role of vanadium, as will be further discussed in the second section.

In the case of vanadium, its redox sensitivity as well its concentration at different pH intervals are crucial in determining its stability [5,6], solubility, and bioavailability, thus determining its direct involvement in several biogeochemical cycles. For this reason, vanadium is considered a suitable redox tracer for the environment as well as for tracking oceanic evolution and atmospheric pollutant transport [7].

Vanadium could have played an important role in the early stages of evolution and life, even considering that it was more readily oxidized from sulfides (together with molybdenum), with respect to the other metals, thus the sea became quite rich in these two elements [8].

Meanwhile, the redox state of vanadium changed from the lower V(III) oxidation state in Archean aqueous geochemistry and mineralogy to higher V(IV) and V(V) states in the Proterozoic and Phanerozoic eras [9].

Another important approach for the study of vanadium oxide activity, within a prebiotic context, could be based on searching for traces of this metal in extra-terrestrial material. Urey-Miller’s experiment first suggested the importance of reducing conditions for the synthesis of biomolecules in primordial Earth’s atmosphere but, then, the following studies led scientists to believe that comets, asteroids, meteorites, and interplanetary dust particles could have provided extra-terrestrial prebiotic organic molecules that, afterwards, combined to generate life-essential molecules, according to the “exogenous delivery hypothesis” [10].

For these reasons, it could be helpful to begin with the study of vanadium compounds in meteorites. The most primitive meteorites (carbonaceous chondrites), in fact, have been found to be very rich of organic molecules of potential importance to the origins of life: sugar acids, hydroxy acids, aldehydes, ketones, amines, amino acids, and nucleobases [11], and it would be interesting to determine if vanadium had played any role in the synthesis of these compounds. In addition, several authors agree that meteorites have played a leading role in the catalytic processes underlying the evolutionary path [12].

As early as the end of 1950s, some studies concerning the determination of metals in rocks and meteorites came out and, among others, neutron activation analysis (NAA) techniques, introduced for the first time by G. Hevesy and H. Levi in 1936 [13], were beginning to be divulged and contributed to a rapid development of this research field. This new type of analysis exploited the newly acquired knowledge about the properties of atomic nuclei, in particular the new concepts of radioactivity, radioactive decay, and nuclear-decay emissions, combined with the discovery of the neutron particle, only four years earlier.

As a result, NAA contributed to the birth of trace elements analyses, allowing the recognition of numerous biologically-essential trace elements and offering a new multi-element method with great applicability in environmental, archaeological, and forensic fields [14].

NAA was used to determine vanadium concentration in several materials (such as graphite, crude oils, biological ashes, and high alloy steels) but no geological application had been reported until 1960, when Kemp and Smales employed this technique to determine the amount of vanadium in five chondritic meteorites [15], with an average value of 65 ppm. It is interesting to take into account that the relatively short half-life of the vanadium isotope formed on neutron-activation (^52^V, 3.76 min) restricted the use of the NAA method to those materials where chemical separation was not necessary.

Either simultaneously and subsequently, other techniques were exploited to analyze vanadium concentration in meteorites, including X-ray fluorescence [16] and the destructive emission spectrographic technique [17].

Once the presence of vanadium in ancient meteorites from which life may originated was demonstrated, it is time to go deeper and try to understand how this metal was involved in the first biological processes and unveil which catalytic activity led to primordial forms of life.

The first example to consider is the possible decomposition of formamide through vanadium oxides. Formamide (HCONH_2_) is the simplest existing amide, which has been found also in comets [18] and interstellar medium [19] and is considered to be an important prebiotic precursor [20,21].

**Figure 1:**
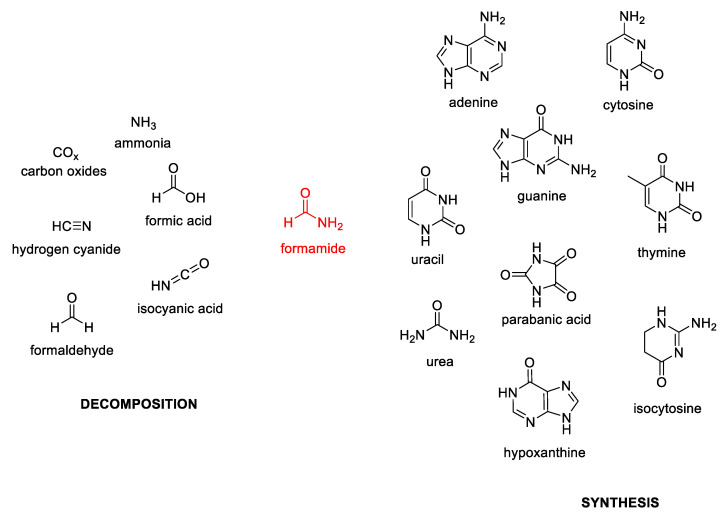
Main decomposition products and synthetic derivatives of formamide.

The peculiarity of formamide is that it condenses into a wide range of nucleic bases and their derivatives, but also into urea, parabanic acid, and carbodiimides, establishing a novel scenario for the origin of biomolecules under plausible prebiotic conditions. The chemistry of formamide can be finely tuned by the presence of a large panel of minerals and metal oxides, acting as catalysts able to promote either its thermal condensation and, at the same time, its partial degradation to lower molecular mass derivatives, including HCN, formaldehyde (HCOH), formic acid (HCOOH), carbon oxides (CO_x_), and ammonia (NH_3_) [22]. All the main decomposition products and synthetic derivatives, arisen from formamide, are reported in Figure 1.

Recently, a theoretical study [23] suggested some decomposition pathways of formamide in the presence of vanadium monoxide and titanium monoxide, including dehydration, decarbonylation, and dehydrogenation, by means of density functional and coupled-cluster theories. Moreover, addition of one or two water molecules in these simulations seemed to catalyze the decomposition, showing lower energy barriers. Direct and indirect formation of urea from formamide was also investigated and results showed that the formation of isocyanic acid (HNCO) in the dehydrogenation pathway represents the first step in the indirect formation of urea from formamide.

At this point, a research issue would be to find where vanadium monoxide was located in the prebiotic universe. Optical spectra of subdwarfs were recorded in 2003 by Lépine, Shara, and Rich [24], showing that VO and TiO are the most important constituents of the atmospheres of these stars; in particular vanadium oxide VO can be generated, in interstellar gas media, in different ways by reaction between vanadium atoms and cationic oxygen species or vanadium cations and oxygen radicals. Furthermore, vanadium nitride (VN) species can also occur in interstellar clouds, obtained from vanadium oxides and the available ammonia formed by a cascade of ion-neutral reactions, starting with the reaction between N^+^ and H_2_. Vanadium nitride is involved in the dehydrogenation of propane to propene and butane to C_4_ olefins, important reactants for the synthesis of unsaturated fatty acids, another class of indispensable biomolecules for life [25].

Vanadium compounds are not only present in the atmosphere of stars or on exoplanets, but can also be found on the Earth′s crust, for example in the form of minerals. These minerals are involved in biomolecule formation and in determining their chirality; chiral crystalline surfaces in fact could have provided effective environments able to discriminate left- and right-handed molecules, determining the prebiotic chiral selection and the organization of essential biomolecules [26].

A huge number of different vanadium-containing minerals have been identified, among which there are: patronite [V^4+^(S_2_^2^^−^)_2_], roscoelite [2K_2_O·2Al_2_O_3_(Mg,Fe)O-3V_2_O_5_·10SiO_2_·4H_2_O], bravonite [(Fe,Ni,V)S_2_], davidite (titanate of Fe,U,V,Cr and rare earths), sulvanite (3Cu_2_S·V_2_S_6_), vanadite [Pb_5_(VO_4_)_3_Cl], and carnotite (K_2_O·2U_2_O_3_, V_2_O_5_·3H_2_O) [27].

Vanadium also occurs in porphyrins and in carbonaceous sedimentary rocks. In porphyrins, VO^2+^ species are strongly coordinated by tetrapyrroles thus resulting in a very stable structure from a thermal point of view. The analyses of oxidovanadium(IV) (VO^2+^) species associated with these macromolecules play an important role in the perspective of this paper to identify possible explanations about the presence and activity of vanadium oxides in the prebiotic era. In order to further investigate this topic, several studies have been carried out about the origin of tetrapyrrole macrocycles in an aqueous environment under mild conditions (in order to mimic prebiotic conditions), starting from α-aminoketones and β-diketones [28,29]. Remarkably, latter compounds acquire, from our point of view, a considerable importance because they are structurally related to the 4-acylpyrazolone ligands, whose origin, biological activity, and coordination ability will be discussed in the third section. Mainly two hypotheses have been conveyed about the nature of VO^2+^ chelating porphyrins; the first one proposes that porphyrins come from chlorophyll and that the inclusion of vanadium happened under extreme reducing conditions after the rock was laid down; the other one, instead, states that porphyrins originated from respiratory pigments of ascidians, a particular marine organisms of which we will discuss later.

So, it is interesting to ascertain if the presence of VO^2+^ porphyrins in these rocks is a testament of the fact that vanadium-based porphyrins were employed in life processes at the time of deposition or, alternatively, these compounds were simply formed from the transformation of other metalloporphyrins. P. I. Premovic. et al. have studied oxidovanadium(IV) porphyrins of the Serpiano marl and the La Luna shaly limestone [30] and the results seem to agree with the second explanation, showing that oxidovanadium(IV) porphyrins were incorporated into the kerogen structure through abiotic geochemical modifications of biosynthetic pigment-chlorophyll. Based on this, Marshall et al. have proposed a new method for the detection of biogenicity of putative microfossil-like structures; the presence of vanadium, in fact, determined through synchrotron X-ray fluorescence imaging, suggests a biological derivation, due to the replacement of Mg^2+^ cations by VO^2+^ in the diagenetic alteration of precursor chlorophyll and heme porphyrin pigment compounds, from living organisms. Therefore, vanadium content, together with microfossil-like morphology and carbonaceous composition, could provide an unambiguous attribution of biogenicity [31].

At the same time, other vanadium-based minerals and inorganic salts have been supposed to be determinant for the evolution of life, acting as active surfaces and concentrating essential biomonomers which compose biomolecules through adsorption and desorption processes on their surfaces, thus allowing their polymerization. In the primordial sea, in fact, divalent transition metal and CN^−^ ions could have reacted, leading to the formation of Fe_4_[Fe(CN)_6_]_3_ complexes and other insoluble metal ferrocyanides, among which were vanadium (II) complexes. These insoluble cyanometal complexes could have settled at the bottom of primordial waters catalyzing on their surfaces a wide range of reactions, among which were the condensation, oligomerization, and oxidation reactions of life’s essential molecules [32].

Another aspect to be considered is vanadium-dependent nitrogenases, which will be discussed later in the paper. Their ability to catalyze both CO and N_2_ reductions has led scientists to believe that these enzymes could have played an important role in the evolution of carbon and nitrogen cycles, suggesting that this property may have been assimilated by ancient microbes through an ancient form of nitrogenase that, subsequently, evolved exclusively towards nitrogen fixation [33].

This hypothesis has been proposed studying the subseafloor microbial habitat associated with hydrothermal vents. The latter are geological fissures on the seafloor, near volcanic areas, that release seawater heated to temperatures even higher than 400 °C by subterranean magma which, during its course through the crust, mobilizes metals from basalt enriching itself of high levels of sulfides obtained from the reduction of sulfate by ferrous iron dissolved in the hot fluid.

Hydrothermal vents are characterized by peculiar geochemical conditions (high temperatures and the presence of several solubilized metal ions) and, for this reason, can host unique animals such as sulfide worms, tubeworms, and ciliates and harbor bacteria with unusual metalloid-associated metabolisms [34].

The solubility of metavanadate and orthovanadate together with the detection of high levels of N_2_ and low values of ammonium and nitrates concentrations in the deep ocean hydrothermal vents has stimulated the search for possible vanadium-based nitrogen fixers which regulate nitrogen cycling, with positive results. Nitrification, the aerobic oxidation of NH_4_^+^ or NO_2_^−^ to nitrate and denitrification have also been observed, suggesting that all the reactions of the nitrogen cycle can occur at hydrothermal vents, probably in different locations of the ecosystem [35].

Certainly, much is still waiting to be discovered about the presence of vanadium oxides in the prebiotic world and about its catalytic activity towards the synthesis of essential biomolecules for life, and many of the inherent mechanisms in these reactions are still rather obscure. Nevertheless, another approach that can be adopted to further investigate the primordial activity of vanadium and its oxides consists of searching for the bioinorganic activity of these compounds inside existing living species, to find out which of these primordial processes have allowed not only life, but also its evolution over millions of years.

## 2. Main Relevant Functions of Vanadium in the Bioinorganic Chemistry

In 2004 Mukherjee et al. highlighted how the bioinorganic role of vanadium compounds is rather contradictory; indeed if, on the one hand, vanadium complexes showed cytotoxicity and aptitude to interfere with different ATPases, protein kinases, ribonucleases, and phosphatases; on the other hand, vanadium deficiency causes several physiological failures affecting, among others, thyroid, glucose, and lipid metabolism [36,37]. This anomaly is further proven by the fact that different organisms bioaccumulate large quantities of vanadium, among which are tunicates [38], fan worms (*Pseudopotamilla occelata*) [39], mushrooms (*Amanita muscaria*) [40], beans (*Pisum sativum*) [41], Alaskan cetaceans, pinnipeds [42], and clams (*Meretrix meretrix*) [43].

Tunicates, also called ascidians or sea squirts, represent the oldest known example of vanadium bioaccumulation in nature and about one hundred years after this discovery, the reason why these organisms bioaccumulate it is still obscure [44]. Someone suggest that vanadium may be involved in oxygen transport and respiration, but it′s not clear why hemocyanin is not enough. Vanadium(V) is stored in its reduced forms either as vanadium(IV) or as vanadium(III) at physiological pH, probably like the hexaaquo complex form in vanadocytes, which are specific vanadium-containing blood cells [45]. The vanadium concentration in the vanadocytes is 0.15 M and achieves 1 M in the vanadophores, vacuoles inside vanadocytes, thus meaning that ascidians are able to concentrate vanadium 10^7^ fold higher than the vanadium concentration in a marine environment [46].

Tunicates, in addition, are the only organisms in which the formation and existence of V(III) in biological systems have been reported, and this is possible thanks to particular peptides called tunichromes; they show a styrylamide structure, probably due to decarboxylation and dehydrogenation of a tripeptide with three trihydroxyphenylalanines.

The process of bioaccumulation [47] is the following: [H_2_VO_4_^−^] from sea water passes into the vanadocytes through the anionic channels of the lipid membrane; subsequently, the [VO_2_]^+^ ion is generated and then it is reduced to the trivalent sulfate vanadium aqua complex, while the peptide is oxidized to quinone which, after decarboxylation, forms styrylamide. The positively charged sulfate vanadium complex cannot pass through the anionic channels and it accumulates in the morula cells, while the tunichrome is able to leave the vanadium-containing vanadocytes.

Vanadium has, among others, two peculiarities that make it active in the biological field:

- The tetrahedral vanadate anion VO_4_^3^^−^ is very similar to the phosphate anion PO_4_^3^^−^ and this allows an interaction with different biological targets that are normally activated by the phosphate itself [48];
- This transition metal can expand its coordination sphere beyond tetrahedral geometry and easily change its oxidation state.

The vanadate and phosphate tetrahedral anions show not only an analogy in structure but also in size, having, respectively, a total volume of 125 and 102 Å^3^ [49]; for this reason, the vanadate anion is able to mimic the phosphate group of enzymes such as phosphatase and kinase. At the same time, however, some important differences emerge: while vanadate forms stable penta-coordinated complexes, for example with ATPases, penta-coordination is only a transition state for phosphate. The state of protonation around neutral pH is also different. Vanadate at pH 7 is present almost exclusively in its deprotonated form (i.e., VO_4_^3^^−^ anion) while phosphate shows both monoprotonated and deprotonated form (i.e., HPO_4_^2^^−^ and PO_4_^3^^−^ anions, respectively), at the same pH.

Many bacteria can use vanadium in various biological functions and its use in bacterial respiration, in which vanadate acts as an electron acceptor, is of particular interest. For example, the facultative anaerobic bacterium *Pseudomonas isachenkovii* reduces vanadate to VO(OH)_2_, by using H_2_ and CO as electron donors [50]. The respiration leads to the production of ATP and the electrons involved in the reduction of vanadate are transported inside the cytosolic membrane to oxidize either the lactate in pyruvate or the formate in carbon dioxide.

In recent years, interest in vanadium compounds has increased, leading researchers to study their activity in various areas. As proof of this, possible applications in the medical field of some vanadium complexes, have been identified [51].

For example, the pharmacological activity of vanadate in improving the symptoms of diabetes is mainly due to the already discussed similarity of this anion with phosphate. Some oxidovanadium (IV) complexes in fact show a response similar to that of insulin, stimulating the absorption and metabolism of glucose through alternative signalling mechanisms [52]. Furthermore, some activity has also been observed in the treatment of parasitic diseases [53], malignant tumours [54] and viral and bacterial infections [55].

The presence of two classes of vanadium-dependent enzymes has been detected in nature: haloperoxidases (VHPO) and nitrogenases (VNases) [56]. The first have been found in marine algae, mushrooms, lichens and cyanobacteria [57] and are directly involved in the use of halides present in waters, especially marine ones, for the synthesis of halogenated organic compounds or, as in the case of chloroperoxidases, in defense of the organism from the attack of bacteria and viruses. The most common substrates of these enzymes are I^−^, Br^−^, and Cl^−^, but sulfides are also oxidized (R_2_S). The oxidant is H_2_O_2_, present in marine waters with a concentration that can reach 0.25 μM. In all these cases, oxidation occurs with the exchange of two electrons.

Vanadium-dependent nitrogenases, on the other hand, catalyze the reduction of N_2_ to NH_4_^+^ (therefore allowing nitrogen fixation, as we have previously seen in the first paragraph), which is coupled with the reduction of H^+^ to H_2_. It is interesting to note that both vanadium and iron-only nitrogenases derive from molybdenum nitrogenase and are produced when the local molybdenum concentration is limited. VNases have been found in diazotrophs among which is *Azotobacter vinelandii*, an obligate aerobic bacterium that shows the peculiarity of containing all three types of nitrogenases (molybdenum, vanadium, and iron-only) [58].

**Figure 2:**
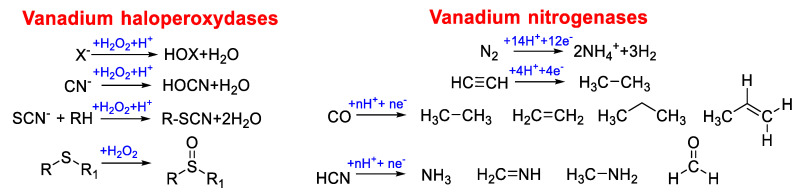
Catalyzed reactions performed by vanadium haloperoxydases and vanadium nitrogenases [39].

This class of metalloenzymes has various similarities with molybdenum nitrogenases in terms of homology in primary sequence and cluster composition since they are composed of a subunit-bridging [Fe_4_S_4_] cluster and one ATP-binding site per subunit (reductase component), and a catalytic component. Nevertheless, peculiar differences exist; even if VNases are less efficient in N_2_ reduction with respect to Mo nitrogenases, they are able to convert ethyne to ethane (ability not shown by Mo counterparts) and reduce CO to hydrocarbons more effectively [59]. The catalytic activity of VHPOs and VNases is reported in Figure 2.

The VHPOs activity may also be connected with problems related to atmospheric processes, in particular to the thinning of the stratospheric ozone level. These enzymes, in fact, catalyze the synthesis of di- and tri-substituted halogen derivatives of methane in the presence of H_2_O_2_ and dissolved organic material, thus forming them as by-products in photosynthetic reactions [60].

Hence, we can focus our attention on the fact that the biological and pharmacological activities carried out by vanadium complexes are certainly a function of the oxidation state of the metal, but also the ligands which chelate the metal centre and play an important role in, for example, affecting the solubility of the organometallic complex or interacting with enzyme pockets through electrostatic and van der Waals interactions.

Previously, we briefly referred to a study suggesting a chemical model for the origin of highly biological relevant tetrapyrrole macrocycles, under prebiotic conditions, that implies the condensation of acyclic dicarbonyl compounds, as β-diketones, and α-aminoketones to form pyrroles which are suitable for successive self-condensation reactions [29].

Therefore, making our choice inside the broad family of chelating ligands capable of providing stable vanadium complexes, the next paragraph will give attention to a class of common ligands for oxidovanadium complexes, within the large family of the β-diketone frame, like acylpyrazolones, highlighting in addition to the chemical properties and the propensity to behave as oxophilic ligands, and the aspects related to their biological/medical activity.

## 3. Structure and Biological Significance of Acylpyrazolones and Their Related Metal Complexes

Acylpyrazolones are a class of β-diketones in which one of the carbonyl moieties is on a chelating arm as substituent of the pyrazolone ring. In 1959, Jensen proposed a new efficient method to synthesize 4-acyl-1-phenyl-3-methylpyrazol-5-ones through condensation reactions of acid chlorides or anhydrides with 1-phenyl-3-methylpyrazol-5-ones, with 70% yields [61].

But is it possible to attribute to pyrazolones a biological/prebiotic origin, if any? With the aim of outlining a likely retro-biosynthetic approach starting from the pyrazolone ring and going gradually back to the various precursors, the first step concerns the formation of 4-hydroxypyrazole (which is in tautomeric equilibrium with pyrazolone) from pyrazole oxidation, catalyzed by cytochrome P-450 IIE1, as it has been observed in rat liver microsomes [62].

Yet, the first naturally occurring pyrazole, β-pyrazol-1-yl-alanine (isomer of histidine), has been observed in *Cucurbitaceae* [63]. Subsequently, after the discovery of 1,3-diaminopropane in *Cucumis sativus,* Brown et al. have individuated this compound as a precursor of the pyrazole moiety and proposed a plausible mechanism for the synthesis of pyrazole by cyclization and dehydrogenation of 1,3-diaminopropane [64]. This latter compound has been demonstrated to be a product of the oxidative cleavage of spermidine [65] which is a polyamine, as well. Polyamines are aliphatic amine metabolites present both in prokaryotic and eukaryotic cells including also putrescine (1,4-diaminobutane) and cadaverine (1,5-diaminopentane) and their role has been associated with a stimulating effect on nucleic acids biosynthesis [66]. The origin of this class of compounds has been linked with arginine and ornithine [67], i.e., aminoacids that, together with citrulline, are involved in the urea cycle, the metabolic cycle occurring in ureotelic organisms in which urea is produced from ammonia. In addition to urea, there is another formamide derivative implied in this cycle: carbamoyl phosphate. Therefore, formamide could represent the last step in this retro-biosynthetic pathway (or, obviously, the first one in the synthetic approach), which makes the connection between pyrazolones and the prebiotic world at least potentially appealing.

**Figure 3:**
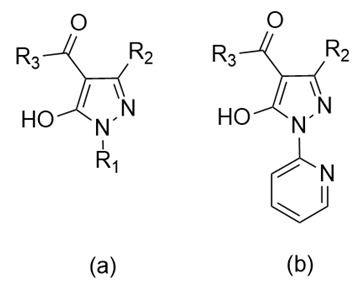
Structure of 4-acylpyrazolones: the placement of 2-pyridyl group in R_1_ position of 4-acylpyrazolones (**a**) introduces a further *N*,*N*-binding site (**b**).

Our interest in the study of acylpyrazoles, as ligands in organometallic chemistry, stems from their structural and electronic characteristics, suitable for a very versatile coordination chemistry, mainly deriving from the simplicity in the functionalization of the pyrazolone ring with different acyl substituents in the C4 position, but also from the coordination ability of the two oxygens, namely those of the acyl moiety and of the enol residue (Figure 3a).

Furthermore, the replacement of the phenyl ring with 2-pyridyl group in the R_1_ position also allowed to obtain ligands with very different coordination skills, as in the example shown in Figure 3b (ambidentate Janus-type ligands).

Initially, acylpyrazolones were studied for their chelating ability toward a wide range of metal ions among which sodium, iron, zinc and even lanthanides [68].

A lot of studies on the solvent extraction of metal ions with acylpyrazolones have been performed; Umetani et al., in particular, investigated the influence of the distance between the two donating oxygens of the ligand on the selectivity for lanthanides ions, confirming that a shorter distance usually corresponds to a better selectivity for metal ions [69]. For this reason, it is an important parameter to take into account in the designing and in the synthesis of acylpyrazolones.

**Figure 4:**
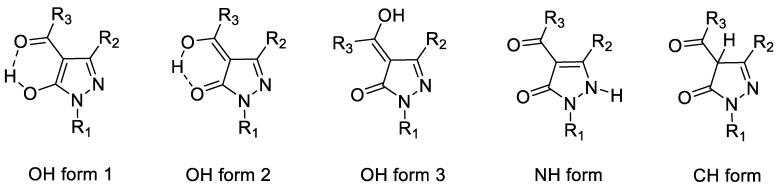
Different tautomeric forms of 4-acylpyrazolones.

Another interesting aspect to be considered concerning acylpyrazolones is their tautomeric versatility: 4-acylpyrazolones in particular present different tautomeric forms which coexist in solution in different ratios, depending on concentration, solvent, temperature, and substituents on the ring, i.e., the OH (to which we refer also with the name of hydroxypyrazoles), NH and CH forms, all reported in Figure 4. ^1^H and ^13^C NMR investigations and X-ray analyses [70] have shown that the OH forms 1 and 2 are predominant in CDCl_3_ and in benzene-*d*_6_, mainly due to the intramolecular hydrogen bonding that stabilizes the structures, while in DMSO-*d*_6_ a significant amount of the NH form is observed.

Afterwards, acylpyrazolones have been tested as versatile ligands for the synthesis of organometallic compounds with catalytic and biological properties, including anti-inflammatory, antioxidant, and antimicrobial activity, but also antiprion, fungicidal, insecticidal, and even anticancer. Moreover, some acylpyrazolones were active against *Mycobacterium tuberculosis* (MTB), which causes tuberculosis, while others exert an inhibitory effect on Tumor Necrosis Factor TNF-α induced expression of ICAM-1 (Intercellular Adhesion Molecule-1), very important for the immune responses and inflammation of human endothelial cells [71].

It should be noted that often the activity of these compounds is not related to the acylpyrazolone itself, but it becomes explicit when they are coordinated with a metal centre. For example, dinuclear (η^6^-arene) ruthenium(II) acylpyrazolone complexes have been tested as anticarcinogens, showing cisplatin-like activity against epithelial carcinoma HeLa and MCF7 tumor cell lines, but a lower cytotoxicity toward normal cell lines [72]. A similar study has been also conducted on platinum complexes with acylpyrazolones as ligands, displaying good results in terms of cytotoxicity against the same tumor cell lines [73].

Vanadium complexes with acylpyrazolones as ligands have also been investigated with the view of finding a biological activity, assuming that many vanadium compounds play an important role in the bioinorganic chemistry of different organisms, as we have already seen in the previous paragraph.

The coordination chemistry of oxidovanadium complexes with acylpyrazolones has been mainly explored for vanadium in the (+4) and (+5) oxidation states. This class of compounds generally shows a distorted square pyramidal geometry for anhydrous compounds, with the two ligands in the equatorial plane in *anti*-configuration and the oxo group in the axial position. When a water molecule is added to the coordination sphere, it will occupy the *trans* position with respect to the oxo group, generating a distorted octahedral environment, even if a few cases in which the water ligand is located in *cis* position are also known [71].

Mixed ligand complexes of oxidovanadium(IV) have been synthesized and tested by Thaker et al. [74] as antibacterial agents against *Staphylococcus* *aureus*, *Escherichia coli*, and *Pseudomonas* *aeruginosa*.

In another paper [75], oxidovanadium (IV) with bidentate heterocyclic azopyrazolone have been shown to be active against human hepatocellular carcinoma (Hep-G2) and human breast carcinoma (MCF-7) cancer cells and one of them showed antibacterial activity against *Escherichia coli*.

These represent further demonstrations of the great versatility of vanadium and its organometallic complexes in terms of biological and catalytic activity underlining, once again, the importance of this element for the purposes of life and its evolution.

Vanadium compounds in recent years have attracted a lot of attention not only for their involvement in biological processes but also, and above all, for their use in oxidative, homogeneous, and heterogeneous catalysis in various industrial reactions. They are able to increase the oxidizing character of various hydroperoxides, including hydrogen peroxide, thus reminding us of the activity that vanadium carries out in vanadium-dependent haloperoxidases, as we have seen previously.

Vanadium complexes, in fact, have shown a great catalytic ability towards several selective oxidation reactions, among which are the epoxidation of alkenes [76], the oxidation of sulfides [77], the oxidative amination [78], the oxidation of alcohols to aldehydes and ketones [79], C–C bond forming reactions [80] and carboxylation [81].

To this end, in the next paragraph some works from our research group related to the study of the catalytic activity of oxidovanadium complexes based on acylpyrazolones ligands will be reviewed, in order to provide a more detailed outline of the richness and versatility of the organometallic chemistry of these types of vanadium complexes.

## 4. Selection of Some Catalytic Applications of Oxidovanadium Complexes from Our Research Group

**Figure 5:**
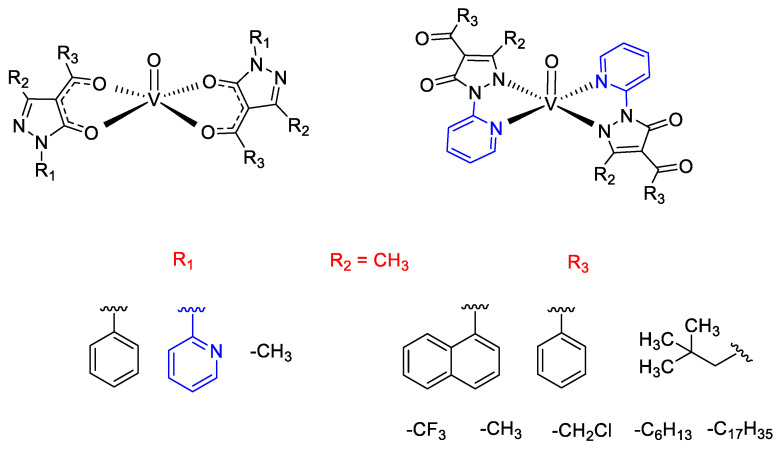
Structures of oxidovanadium complexes based on 4-acylpyrazolones ligands whose catalytic activity has been studied by our research group.

Initially, our interest focused on the synthesis of oxidovanadium complexes with acylpyrazolones ligands in which the substituent groups in R_1_ position are a phenyl, 2-pyridyl, or methyl moiety while the substituents in R_3_ are neopentyl, methyl, trifluoromethyl, phenyl, or 1-naphthoyl (the structures of vanadium complexes are reported in Figure 5, the R_2_ position being in all cases occupied by a methyl group). These complexes were synthesized and fully characterized by elemental analyses, IR, ESI-MS, electron spectroscopy, magnetic susceptibility measurements and, in some cases, by EPR spectroscopy and X-ray diffraction too. Afterwards, they were tested as homogeneous catalysts in the selective oxidation of styrene, α-methylstyrene, and cis-β-methylstyrene, in the presence of H_2_O_2_ as the primary oxidant, under mild conditions [82].

Solvent, temperature, catalyst amount, and oxidant concentration have been evaluated in order to optimize both activity and selectivity of the oxidative reactions. Results have shown high values of conversion of substrates (81–98%) and highlighted how, generally, shorter reaction times (4 h) lead to higher selectivity, but at the same time lower substrate conversions.

**Figure 6:**
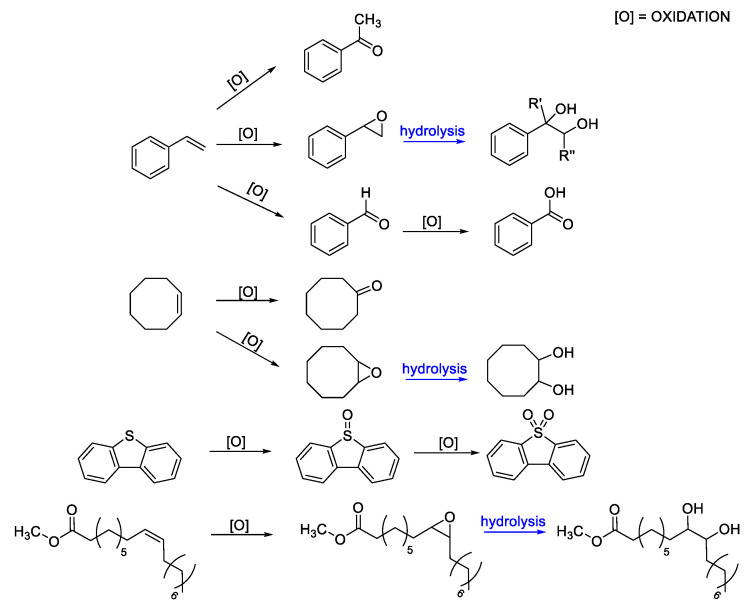
Schematic representation of the main substrates and their oxidation products analyzed by our research group.

In Figure 6, a selection of substrates and their corresponding oxidation products observed during our catalytic studies, with vanadium complexes bearing 4-acylpyrazolones ligands, are reported.

**Figure 7:**
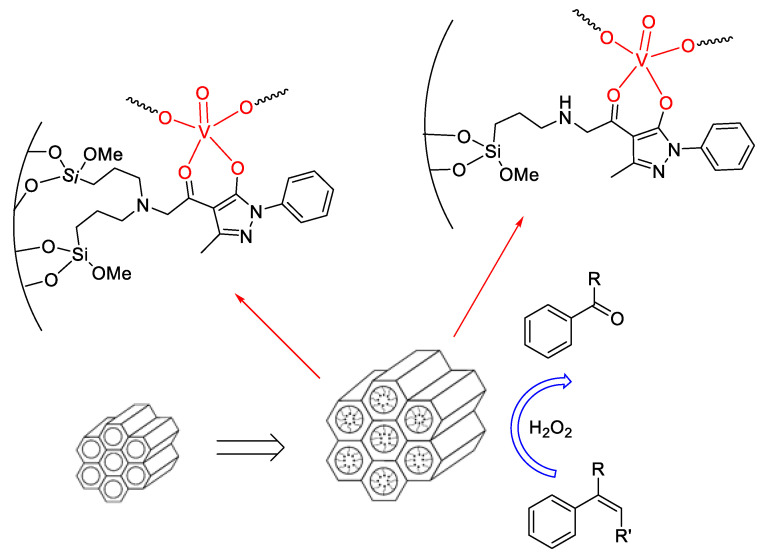
Sketch of the general procedure for the preparation of SBA-15 anchored oxidovanadium based catalysts.

Once the catalytic activity of oxidovanadium complexes based on acylpyrazolonate ligands was tested, in homogeneous conditions, we then moved toward the investigation of the heterogeneous catalysis. To this end, we functionalized a highly stable mesoporous silica support, SBA-15 (Santa Barbara Amorphous-15) with aminopropyl chains, through a post-synthetic grafting method, thus allowing it to covalently bind proper ligands based, among others, on acylpyrazolone units in order to obtain, after the loading of oxidovanadium(IV) precursors, new vanadium containing “quasi-homogeneous” catalysts [83]. In addition, the same amino functionalized SBA-15 support was used for the anchoring of a previously preformed oxidovanadium(IV) complex, containing acylpyrazolone ligands, properly functionalized with a chloromethyl unit, within the acyl moiety, in order to allow the formation of a covalent bond, after reaction with the amino group (Figure 7).

Successively, these anchored catalytic systems have been fully characterized and studied for the selective oxidation of conjugated olefins. Very high selectivity towards the formation of benzaldehyde (starting from styrene and β-methyl styrene) or acetophenone (starting from β-methyl styrene) has been disclosed and, very interestingly, the selectivity of the heterogeneous catalysts showed to be even higher than that displayed by their homogeneous analogues, previously studied.

Moreover, theoretical DFT (Density Functional Theory) calculations on the probable mechanistic pathways involved in the oxidation of ethylene and 1,3-butadiene (as model substrates chosen, respectively, for simple and conjugated olefins) promoted by the oxidovanadium(IV) acetylacetonate [VO(acac)_2_] complex, have confirmed the experimental results we previously described, in terms of the observed selectivity toward different products formation, arisen from the competition pathways between epoxidation and oxidative cleavage of starting materials [84]. Indeed, theoretical results showed quite different scenarios depending on the conditions employed for the oxidation, confirming that, under kinetic control, in both the model substrates, the double bond oxidative cleavage is always the favored path, although in the case of 1,3-butadiene the difference with the epoxidation route is enhanced, thus confirming that the selectivity is sensitive to the nature of the substituent in the vinyl position.

**Figure 8:**
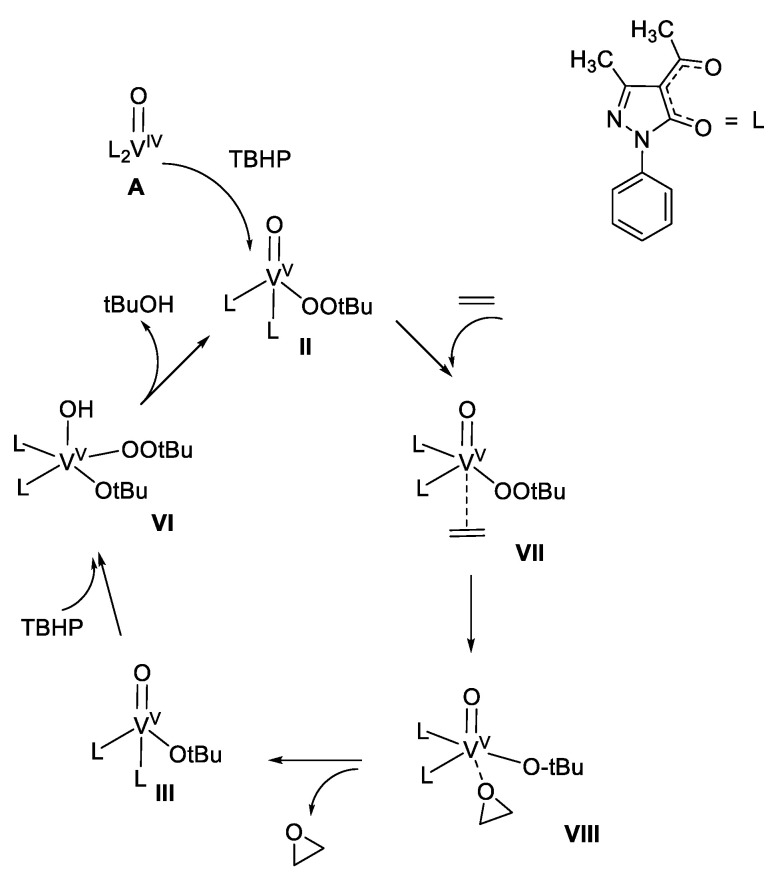
Sketch of the likely catalytic cycle operative under the TBHP oxidation promoted by oxidovanadium complex **A**.

Thereafter, we have tested other similar oxidovanadium complexes (based on acylpyrazolones ligands having a phenyl group in R_1_ position, a methyl in R_2_ while the substituents in R_3_ are methyl, trifluoromethyl or -CH_2_Cl, respectively, as outlined in Figure 5), with the aim to investigate them as catalysts for the selective and mild oxidation of unsaturated fatty acid methyl esters (FAMEs), in particular methyl oleate (C18:1), methyl linoleate (C18:2), and methyl linolenate (C18:3) [85]. *Tert*-butylhydroperoxide (TBHP) has been used as main oxidant and catalytic reactions, performed with or without solvents, have revealed high conversions of starting materials and high selectivities in the formation of corresponding mono- di- and tri-epoxides, especially under solvent-free conditions. Interestingly, in this paper the ESI–MS investigation of a FAME model substrate (methyl 4-pentenoate) in the presence of the oxidovanadium catalyst containing the methyl group in the acyl fragment, allowed us to propose a likely catalytic cycle operative under the TBHP promoted oxidation, where the transient oxidovanadium(V) complex containing the *tert*-butylperoxyl group (**II**), formed from the starting complex **A** after the initial oxidation, may have a key role (Figure 8).

Recently, we have investigated the structural and catalytic study of two novel oxidovanadium complexes based on acylpyrazolones ligands with different chain lengths in the acyl moiety (6 and 17 carbon atoms, respectively, see Figure 5), exploring either the oxidation of sulfides to their corresponding sulfones or the oxidation of styrene and *cis*-cyclooctene, having in mind to compare their activity both in conventional solvents and ionic liquids [86].

We have chosen dibenzothiophene (DBT) and 4,6-dimethyldibenzothiophene (DMDBT) as models of organic sulfur compounds contained in diesels, in a view of potential application of these new catalysts for the oxidative desulfurization (ODS), i.e., the industrial process employed for the achievement of ultra-low-sulfur diesels. The results of this work have exhibited high conversion of substrates and very high selectivity, especially in the case of DBT with TBHP as an oxidant in which the selectivity towards the formation of sulfone is always 100%, independent of the solvent employed. DFT studies confirmed the observed better experimental activity of the two catalysts in the presence of TBHP as oxidant in comparison to H_2_O_2_, for all the substrates. Finally, preliminary calculations carried out for roughly modeling what might happen when ionic liquids (IL) are used, suggested that the starting VO(Q^Me^)_2_ complex, chosen as a model, might undergo a partial or complete disrupture, essentially due to the strong ionic interaction among oxidovanadium(IV) cation and ionic liquid anion. This observation might qualitatively explain the substantially not so relevant efficiency observed in ILs, in comparison to conventional solvents. On the other hand, it must be evidenced that the system formed by oxidovanadium complex having the C6 alkyl chain in the acyl moiety (Figure 5), and BMIM-PF_6_, used for the study of DBT oxidation with TBHP, showed to be stable, retaining the activity of the catalyst caught inside the ionic liquid phase and so allowing its recyclability, without a relevant catalyst leaching, at least for five running cycles.

## 5. Conclusions

In this report, starting from the most updated theories on the role of vanadium oxides in prebiotic chemistry and trying to unravel their central position, if any, in the primordial chemistry of formamide, we briefly outlined a description of the main relevant functions of vanadium in biological inorganic chemistry. With a certain emphasis we placed our attention, in particular, on its capability, as the oxidovanadium(IV) ion, to act as a catalytic promoter in many selective oxidation processes, after binding with versatile ligands such as the chelating and biologically relevant acylpyrazolones. A conceivable retro-biosynthetic pathway that potentially connects the pyrazolone framework to formamide, which is one of the most likely prebiotic chemical precursors of biologically-relevant compounds, has also been briefly sketched.

Although the role that vanadium could have actively played in a prebiotic evolutionary world has not yet been clearly demonstrated, nevertheless the various hypotheses on prebiotic chemistry which have been so far proposed can help to better outline the contours within which delineate and define in a more realistic way this role. Main dissertations in the context of life occurrence in the prebiotic world, range from a terrestrial perspective, for example, the active role deriving from the particular high temperature and pressure values present in hydrothermal vents [87], to an extraterrestrial one, e.g., the several biogenic functions attributed to meteorites [12].

In addition, the diverse postulates conceived on prebiotic chemistry, have frequently underlined the key functions played by metals (starting from the primordial Fe–S proteins to the most sophisticated architectures of the several types of metalloproteins), as essential catalytic agents in promoting synthetic reactions for the formation of the most complex biological polymers. The latter formed starting from simple chemical molecules such as HCN, NH_3_, HCHO, H_2_O, etc. For these reasons, the rich and diversified chemistry shown by vanadium derivatives and their proven role in biological inorganic chemistry prompted the scientific community to continue its efforts in unveiling of one or more possible mechanisms by means of which this fascinating element could have played an essential function, at least in the early stages of life on Earth.
